# Bridging anatomy and function in ischemic VT: CT wall-thickness and coherent mapping insights

**DOI:** 10.1007/s10840-026-02333-7

**Published:** 2026-04-22

**Authors:** Kivanc Yalin, Hakan Yalman, Ali Ugur Soysal, Sebnem Durmaz, İlayda Bilgili Altuntas, Baris İkitimur, Ahmet Kaya Bilge, Kazim Serhan Ozcan, Henry D. Huang, Tolga Aksu

**Affiliations:** 1 Cerrahpasa Faculty of Medicine, Department of Cardiology, Istanbul University Cerrahpasa, Istanbul, Turkey; 2 Cerrahpasa Faculty of Medicine, Department of Radiology, Istanbul University Cerrahpasa, Istanbul, Turkey; 3 Cerrahpasa Faculty of Medicine, Department of Anesthesiology and Reanimation, Istanbul University Cerrahpasa, Istanbul, Turkey; 4 Atakent Hospital, Acibadem University, Cardiology Clinic, Istanbul, Turkey; 5 Dr. Siyami Ersek Hospital, Cardiology Clinic, Istanbul, Turkey; 6 Department of Cardiology, Chicago University, Chicago, IL USA; 7 Department of Cardiology, Istanbul Aydin University, Istanbul, Turkey; 8 Medical Park Florya Hospital, Department of Cardiology, Istanbul Aydin University, Istanbul, 34742 Turkey

**Keywords:** Ventricular tachycardia, Ischemic cardiomyopathy, Coherent mapping, Isochronal mapping, Computed tomography

## Abstract

**Background:**

Functional mapping tools such as Coherent Mapping (CM) and Isochronal Late Activation Mapping (ILAM) allow visualization of conduction slowing and block during ventricular tachycardia (VT) ablation; however, their anatomical correlates within computed tomography (CT)–derived myocardial wall-thickness (WT) maps are incompletely defined. We aimed to evaluate the spatial relationship between CT-derived WT characteristics and functional electroanatomical markers and to assess the predictive value of WT-derived conduction channels (WTC) for identifying critical VT isthmuses.

**Methods:**

Seven consecutive patients with ischemic cardiomyopathy undergoing VT ablation and pre-procedural contrast-enhanced CT were analyzed. WT maps and WTCs were reconstructed using the inHeart™ platform and integrated with CARTO 3 electroanatomical maps. CM-SCZs, ILAM-DZs, and lines of blocks (LOBs) were identified using predefined criteria and projected onto WT models. Local WT values, overlap with WT ≤ 5 mm regions, and Dice similarity coefficients were calculated to quantify anatomical–functional concordance.

**Results:**

Eighteen CM-SCZs, 15 ILAM-DZs, and 12 LOBs were identified. Functional regions predominantly localized within thinned myocardium (WT ≤ 5 mm). Mean WT was 3.6 ± 0.8 mm for SCZs, 3.8 ± 0.9 mm for DZs, and 4.2 ± 1.1 mm for LOBs, compared with 6.3 ± 1.3 mm in remote myocardium (*p* < 0.01). SCZs overlapped with WT ≤ 5 mm areas by 74 ± 12% (Dice 0.67 ± 0.10), while DZs showed 70 ± 15% overlap (Dice 0.63 ± 0.11). Direct correspondence between WTCs and SCZs occurred in 50% of cases.

**Conclusions:**

Although CT-derived WTCs identify regions at risk, functional mapping remains essential for precise substrate delineation. Integrated CT and CM/ILAM analysis may enhance targeting efficiency during VT ablation.

**Graphical Abstract:**

**Figure:**
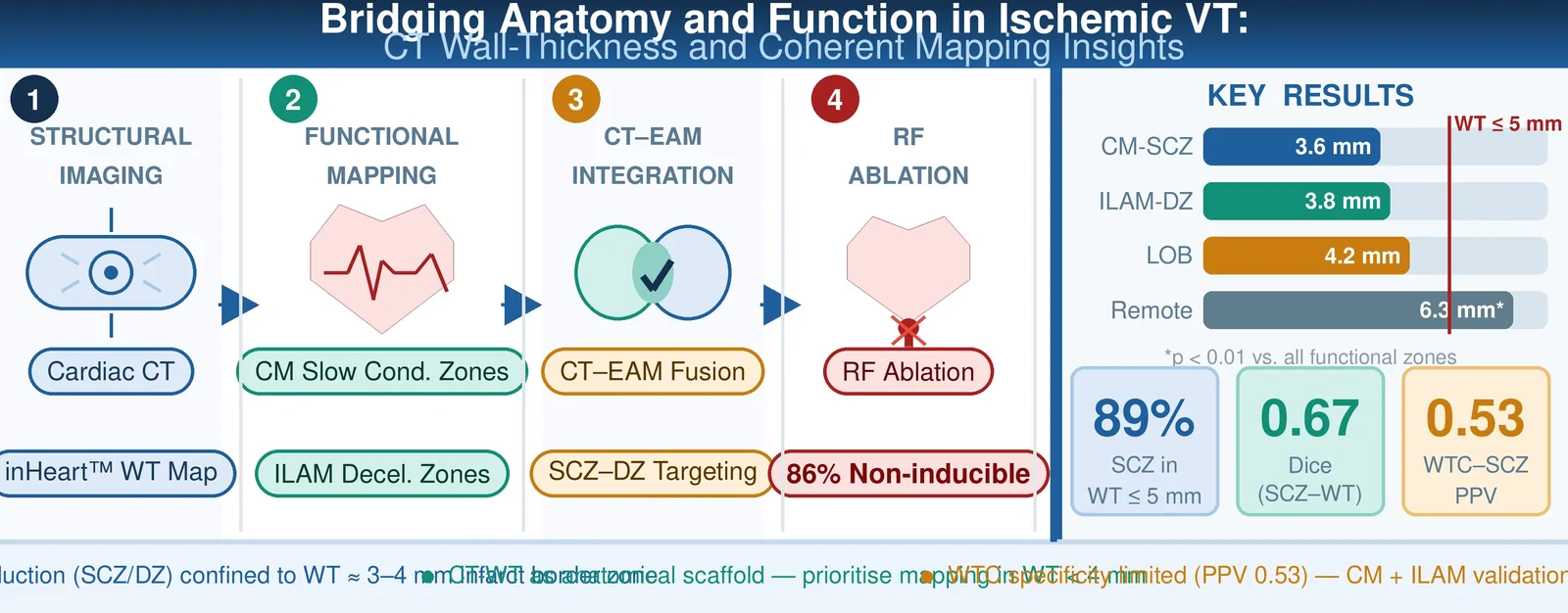

## Introduction

Ventricular tachycardia (VT) represents a serious and potentially fatal arrhythmia most commonly seen in the setting of ischemic cardiomyopathy (ICM), where the myocardium has undergone extensive scarring. The presence of fibrotic tissue distorts the normal electrical architecture of the heart, producing areas of non-uniform conduction and slow electrical propagation. These heterogeneous regions form the substrate for reentrant circuits, the principal mechanism responsible for sustaining VT. Therefore, precise identification and ablation of these critical conduction pathways are essential to prevent recurrence of this life-threatening arrhythmia [1–3]. Despite advances in electroanatomical mapping, defining these critical pathways remains challenging, particularly in cases of hemodynamic instability or non-inducible VT. Recently, novel functional mapping tools such as Coherent Mapping (CM) and Isochronal Activation Mapping (ILAM) have provided improved visualization of activation slowing and conduction block, offering a dynamic representation of the reentrant substrate beyond conventional voltage or late-potential maps [4, 5].

Parallel to these developments, cardiac computed tomography (CT) has become a valuable modality for the structural characterization of post-infarction myocardium. Quantitative wall-thickness (WT) maps derived from CT datasets can delineate regions of transmural scarring and identify WT-derived conduction channels (WTCs) that anatomically correspond to the infarct border zone [6–8].

However, while most arrhythmogenic areas lie within thinned myocardium, the predictive value of these CT-based channels for functional slow conduction remains limited. Takigawa et al. demonstrated that although all VT isthmuses were contained within CT-defined channels, only about half of the channels actually harbored critical conduction pathways [6]. Similarly, Maher et al. showed that in ICM patients, > 90% of functional deceleration zones were located in regions with WT ≤ 5 mm, but that the spatial overlap between CT thinning and true slow-conduction corridors remained partial [7]. In addition, Yamashita et al. reported that myocardial wall thinning measured by MDCT predicts transmural scar and arrhythmogenic substrate, supporting the use of WT mapping as a marker of structural remodeling [8].

Building on our previous work characterizing CM-SCZs, ILAM-DZ and LOB within low-voltage and border zones of ischemic scars [4], the present study integrates these functional descriptors with WT maps generated using the inHeart™ platform.

The objectives were to:

1. quantitatively assess the spatial relationship between CM-SCZs, ILAM-DZs, LOBs, and local myocardial WT;
2. determine the degree of anatomical–functional concordance between WTC and electrophysiologically verified slow-conduction corridors; and.
3. explore whether anatomical information obtained from WT analysis could help define which myocardial regions warrant more concentrated functional mapping during substrate characterization.

We hypothesized that identifying a consistent relationship between anatomical WT features and functional substrate regions could support the potential for using structural imaging either as a complementary tool or, when sufficient concordance exists, as a surrogate framework for substrate delineation.

## Materials and methods

### Study population

This study included seven consecutive patients with ischemic cardiomyopathy (ICM) who underwent catheter ablation of scar-related ventricular tachycardia (VT) at our institution between September 2024 and August 2025 and had available pre-procedural contrast-enhanced cardiac CT datasets suitable for wall-thickness analysis. Inclusion criteria comprised [1] structural heart disease due to prior myocardial infarction, [2] sustained monomorphic VT documented clinically or induced during electrophysiological testing, and [3] availability of complete electroanatomical and imaging data. Exclusion criteria included non-ischemic cardiomyopathy, severe renal dysfunction (eGFR < 30 mL/min/1.73 m²), or inadequate image quality for CT segmentation. The institutional ethics committee approved the study protocol, and all patients provided written informed consent.

### Cardiac CT acquisition and wall-thickness analysis

Pre-procedural cardiac CT scans were performed on a 64-slice multidetector scanner (Somatom Force, Siemens Healthineers) using ECG-gated contrast enhancement. Typical parameters were: 120 kVp, 0.6 mm slice thickness, and end-diastolic reconstruction at 75% of the R–R interval.

The datasets were processed using the inHeart™ platform (inHEART, Bordeaux, France), which provides semi-automated segmentation of the left ventricular endocardial and epicardial contours and generation of a three-dimensional wall-thickness (WT) map. WT was computed as the minimal distance between corresponding endocardial and epicardial surfaces. Areas with WT ≤ 5 mm were defined as thinned myocardium, and WT-derived conduction channels (WTCs) were automatically extracted as linear corridors of thinned tissue bordered by thicker myocardium, following previously published criteria [6–8].

Quantitative channel parameters—including length, width, mean WT, minimal WT, and orientation relative to the mitral annulus—were exported for analysis. All CT-derived meshes were rigidly registered to the CARTO 3 electroanatomical maps using fiducial anatomic landmarks (aortic root, mitral annulus, LV apex), with a surface-to-surface registration error ≤ 3 mm.

High-density substrate mapping was performed using the CARTO 3 system (Biosense Webster, Irvine, CA) with either a Pentaray or Octaray catheter. Mapping was conducted during sinus rhythm in six patients and during right ventricular pacing in one. Mapping methodology, voltage definitions, and conduction analysis criteria for Coherent Mapping (CM) and Isochronal Late Activation Mapping (ILAM) followed the standardized protocols described in our previous work [4].

To ensure reliable functional interpretation despite inter-patient variability in map density, all maps met a predefined minimum density threshold (≥ 1000 points). In segments where point density was relatively lower—typically within dense-scar regions—electrograms were manually reviewed, and approximately 5% of points required reannotation due to multicomponent or far-field signals. Functional zones including SCZs, DZs, and LOBs were accepted only when their boundaries demonstrated stability across both CM vector fields and ILAM isochronal contouring, minimizing the influence of local point sparsity and ensuring consistent comparison with CT-derived wall-thickness features.

Programmed ventricular stimulation was delivered from the right ventricular apex using a 500-ms drive train and up to three extrastimuli, with a minimum coupling interval of 240 ms. When hemodynamically tolerated, induced VTs were activation-mapped and targeted directly.

Radiofrequency (RF) ablation was performed using an irrigated 3.5-mm tip catheter (ThermoCool SmartTouch, Biosense Webster) with settings of 35–45 W and 17–30 mL/min irrigation flow. The ablation strategy prioritized functional mapping guidance, specifically targeting SCZ–DZ overlap regions, terminal activation corridors, and the terminations of LOBs. In cases where VT was not tolerated, substrate modification was performed within low-WT border zones exhibiting fractionated or late potentials and CM vector thickening on the fused maps. Procedural endpoints included the elimination of local abnormal ventricular activity and non-inducibility of any monomorphic VT after a 30-minute waiting period.

### Integration of CT and functional maps

The CT-derived WT models were imported into CARTO 3 via the inHeart export interface. Fusion accuracy was verified visually and quantitatively (surface error < 3 mm). Functional regions (CM-SCZs, ILAM-DZs, LOBs) were projected onto the WT map to assess spatial correspondence.

For each zone, the following parameters were computed:

(i) Local mean WT (mm).
(ii) Minimal distance (mm) to nearest WT ≤ 5 mm area.
(iii) Overlap percentage (%) between functional area and WT ≤ 5 mm.
(iv) Dice similarity coefficient (DSC) between CM-SCZ / ILAM-DZ masks and WT ≤ 5 mm masks.
(v) WTC correspondence (%): proportion of functional areas traversing a CT-defined channel.

### Statistical analysis

Continuous variables were expressed as mean ± standard deviation or median (interquartile range), as appropriate. Normality was assessed using the Shapiro–Wilk test.

Comparisons of wall thickness (WT) between functional (CM-SCZ, ILAM-DZ, LOB) and non-functional myocardial regions were performed using the paired t-test or Wilcoxon signed-rank test, depending on data distribution.

Categorical variables were compared using the Fisher’s exact test. Statistical tests were conducted using SPSS version 29 (IBM, Chicago, IL) for hypothesis testing. The Dice similarity coefficient between functional and anatomical masks was calculated using a custom Python-based script (NumPy/SciPy environment) after spatial registration of the CT and EAM meshes.

### Clinical and procedural details (CT-WT subset)

A total of 7 patients with ischemic cardiomyopathy (ICM) who underwent VT ablation with pre-procedural CT-based WT analysis were included in the imaging subset. Clinical characteristics were summarized in Table 1. Substrate mapping was performed endocardially in all patients, whereas in one case an additional epicardial approach was undertaken owing to anterior scar extension suggesting epicardial involvement. Mapping rhythm was sinus rhythm in six patients and right ventricular pacing in one. The mean number of mapping points in substrate maps was 2210 ± 1480, while a VT activation map was available in one patient, comprising 278 points. Manual reannotation was required in approximately 5% of substrate points, predominantly in areas with fractionated or multicomponent electrograms, where far-field annotation errors were corrected. Radiofrequency ablation targeted the critical isthmus or late-potential clusters identified by Coherent Mapping (CM) and Isochronal Activation Mapping (ILAM), achieving non-inducibility of any clinical VT in six patients (86%) at the end of the procedure. There were no major procedural complications, and all patients were discharged on optimized guideline-directed medical therapy.

**Table 1:** Baseline clinical characteristics

Parameter	Total (*n* = 7)
Age, years	64.7 ± 8.9
Male sex	6 (85.7%)
Diagnosis	Ischemic cardiomyopathy (7, 100%)
Left ventricular ejection fraction, %	33 ± 7
Prior myocardial infarction	5 (71%)
ICD implanted	7 (100%)
Appropriate ICD therapy prior to ablation*	7 (100%)
Hypertension	5 (71%)
Diabetes mellitus	2 (29%)
Chronic kidney disease (stage 2–3)	2 (29%)
Beta-blocker therapy	7 (100%)
Amiodarone therapy (pre-procedural)	6 (86%)

### Quantitative analysis of the CT–derived wall thickness subset

Among the study population, a subset of 7 patients underwent pre-procedural contrast-enhanced cardiac CT segmentation using the inHeart™ platform to generate individualized myocardial wall-thickness (WT) maps and wall-thickness–derived channels (WTCs). These cases were selected based on the availability of high-resolution CT datasets acquired within three months prior to the ablation procedure and the presence of extensive scar tissue on electroanatomical mapping.

The integration of WT models with CM and ILAM was successfully achieved in all patients, enabling direct spatial correlation between anatomical and functional substrate descriptors. Detailed activation mapping could be performed in only one hemodynamically stable VT case, whereas in the remaining patients, analysis was based on substrate mapping. Region-based comparisons between CM-SCZ, ILAM-DZ, LOB, and the corresponding local wall-thickness values were subsequently performed.

Initial inspection demonstrated that most functionally abnormal areas were confined to regions of myocardial thinning within the infarct border zone, typically ≤ 5 mm WT, whereas preserved-thickness segments (> 7 mm) were rarely associated with slow conduction. WTCs, defined as narrow corridors of relative wall thinning bordered by thicker myocardium, were identifiable in all patients and showed topographical proximity to critical conduction pathways delineated by CM vectors.

**Fig. 1:**
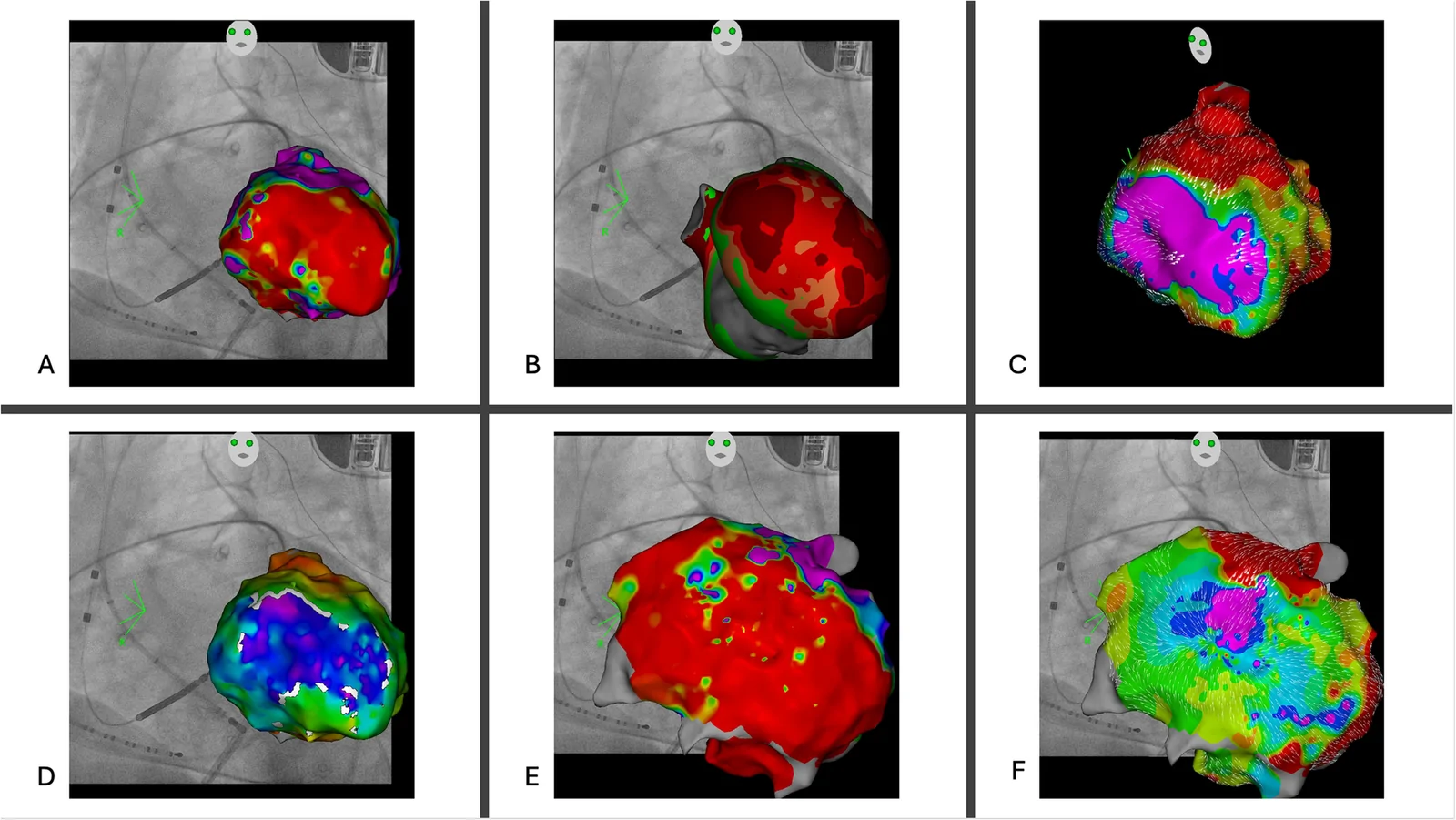
Representative case demonstrating anatomical–functional integration of endocardial and epicardial findings with CT wall-thickness mapping. Panel **A** shows the endocardial voltage map, where red areas indicate regions with voltage < 0.5 mV. Panel **B** displays the CT-derived wall-thickness (WT) map, with darker shades representing thinned myocardium. Panel **C** demonstrates that both ILAM-derived deceleration zones (DZs) and Coherent Mapping–identified slow conduction zones (CM-SCZs) are localized within myocardial regions measuring 2–3 mm in thickness. Panel **D** highlights lines of block (LOBs) marked in white. Panel **E** shows the corresponding epicardial voltage map of the same patient, revealing an extensive low-voltage area, while Panel F depicts epicardial DZs co-localizing with CM-SCZs in the same region

**Fig. 2:**
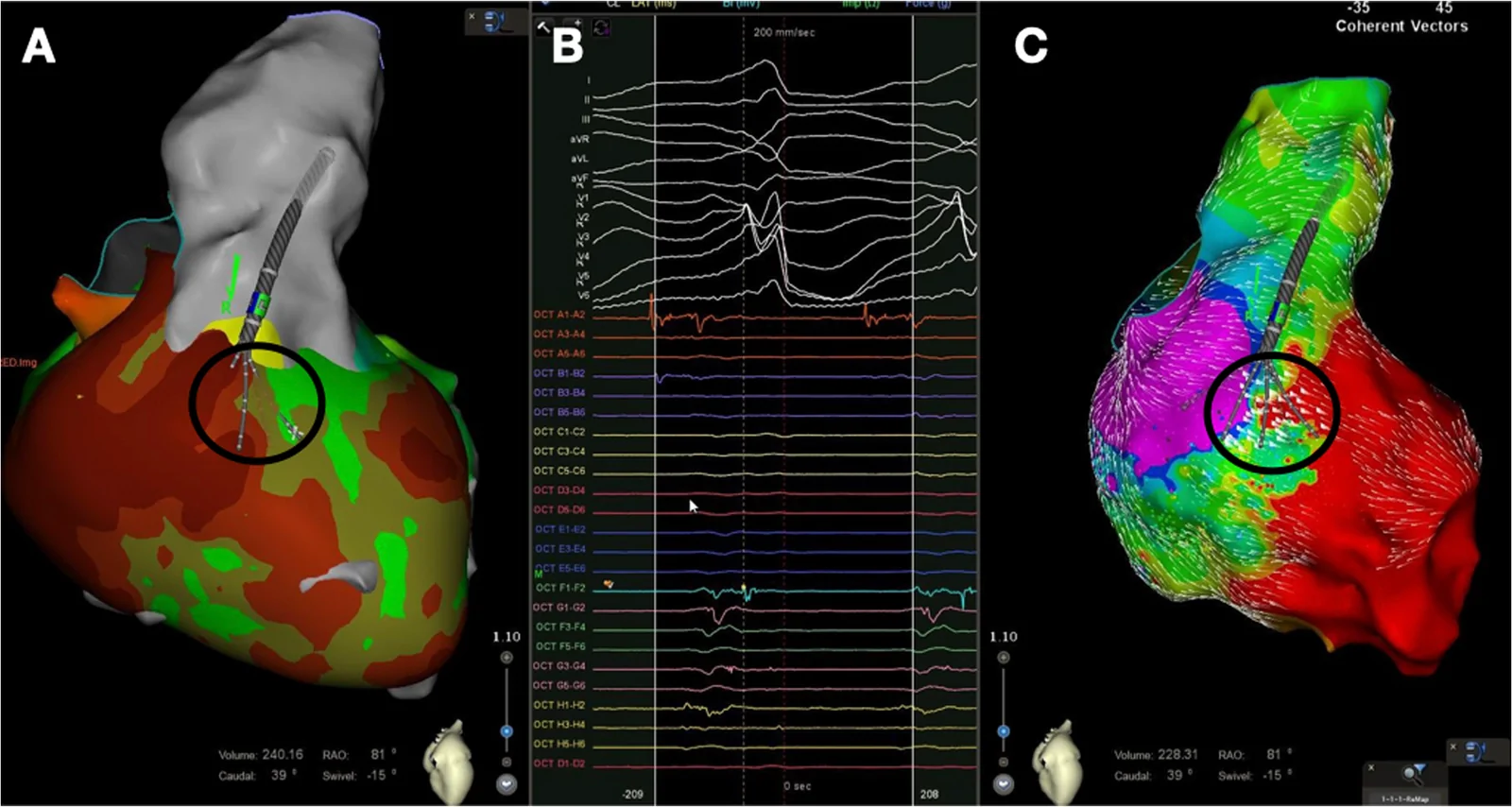
Integration of CT-derived wall-thickness (WT) and functional electroanatomical findings in a patient with post-infarction VT. Panel **A** shows the fusion of the endocardial voltage map with the CT-based WT model, demonstrating spatial correspondence between low-voltage areas (red) and regions of myocardial thinning. Panel **B** displays intracardiac electrograms recorded during VT, highlighting mid-myocardial potentials within the scar region. Panel **C** depicts the overlap of the deceleration zone (DZ) identified by Isochronal Late Activation Mapping and the slow conduction zone (SCZ) defined by Coherent Mapping. VT termination occurred during ablation at this site, which is marked by the black circle

Detailed quantitative results regarding overlap ratios, minimal distance measurements, and wall-thickness gradients between each mapping parameter are summarized in Table 2 and illustrated in Figs. 1 and 2.

**Table 2:** Quantitative relationship between functional mapping parameters and CT-derived wall-thickness metrics

Parameter	No. of zones (*n*)	Mean WT (mm)	% Located in WT ≤ 5 mm	Mean Distance to WT ≤ 5 mm (mm)	% Overlap with WT ≤ 5 mm	WTC Sensitivity / PPV	Dice Index (%) †
CM-SCZ	18	3.6 ± 0.8	88.9	2.3 ± 1.6	74 ± 12	0.62 / 0.53	67 ± 10
ILAM-DZ	15	3.8 ± 0.9	86.7	2.6 ± 1.9	70 ± 15	0.59 / 0.49	63 ± 11
LOB	12	4.2 ± 1.1	75.0	3.0 ± 1.5	61 ± 14	0.48 / 0.41	58 ± 9

This table summarizes the spatial correlation between functional electroanatomical markers—CM-SCZ, ILAM-DZ, and LOB—and anatomical WT parameters derived from pre-procedural cardiac CT. Most functional abnormalities were confined to myocardial regions with WT ≤ 5 mm, exhibiting high overlap with areas of wall thinning. *WTC* wall-thickness-derived conduction channel, *PPV* positive predictive value. †Dice index represents the spatial similarity coefficient between functional and anatomical masks

#### Spatial relationship between CM-SCZ, ILAM-DZ, and wall thickness

A total of 18 CM-SCZ, 15 ILAM-DZ, and 12 LOB were identified in the 7 patients included in the CT–wall-thickness subset.

When projected onto the CT-derived WT maps, 16 of 18 CM-SCZs (88.9%) and 13 of 15 ILAM-DZs (86.7%) were located within or immediately adjacent to regions with WT ≤ 5 mm. The mean wall thickness measured within CM-SCZs was 3.6 ± 0.8 mm, within ILAM-DZs 3.8 ± 0.9 mm, and along LOBs 4.2 ± 1.1 mm, compared with 6.3 ± 1.3 mm in remote myocardium (*p* < 0.01 for all comparisons).

The mean minimal distance between the center of CM-SCZs and the nearest WT ≤ 5 mm region was 2.3 ± 1.6 mm, while for ILAM-DZs this value was 2.6 ± 1.9 mm. Overlap percentage between CM-SCZs and WT ≤ 5 mm areas averaged 74 ± 12%, and Dice similarity coefficient was 0.67 ± 0.10; corresponding values for ILAM-DZs were 70 ± 15% overlap and 0.63 ± 0.11 Dice index.

LOB most frequently followed the interface between WT ≤ 4 mm and WT > 6 mm zones, with mean WT gradient of 2.1 ± 0.7 mm/mm, and were spatially contiguous with at least one CM-SCZ in 9 of 12 cases (75%).

WT-derived conduction channels (WTCs) were detected in all patients (median 3 per patient, range 2–5). However, direct correspondence between WTCs and CM-SCZs was observed in only 9 of 18 CM-SCZs (50%), yielding a positive predictive value (PPV) of 0.53 and sensitivity of 0.62 for identifying functionally confirmed slow-conduction corridors.

In the single patient with hemodynamically tolerated VT, the activation map enabled direct correlation of tachycardia propagation with anatomical and functional findings. The critical isthmus demonstrated complete convergence of CM-defined slow conduction, ILAM-derived deceleration, and a region of myocardial wall thinning ≤ 4 mm on CT. This case provided unique mechanistic validation of the integrated anatomical–functional approach by showing that the VT circuit localized precisely to the overlap of functional SCZ/DZ and structurally thinned myocardium.

## Discussion

### Principal findings

In this study, we integrated pre-procedural CT–derived wall-thickness (WT) analysis with high-density electroanatomical mapping using CM and ILAM in patients with ischemic cardiomyopathy (ICM). The main findings can be summarized as follows:

1. Functional conduction abnormalities—including CM-SCZs, ILAM-DZs, and LOBs—were predominantly localized within moderately thinned myocardial segments with WT values between 3 and 4 mm.
2. Despite this clear anatomical–functional correspondence, the agreement between WTCs and functionally verified conduction corridors was modest, with only approximately half of CM-SCZs colocalizing with a WTC.
3. These results indicate that while myocardial thinning is a prerequisite for slow conduction, structural information alone is insufficient to delineate ablation targets precisely.

Together, these findings suggest that WT mapping provides a useful anatomical framework for identifying regions at risk, but functional mapping remains indispensable for precise substrate definition and procedural guidance. Importantly, even in maps with modest point density, functional abnormalities remained tightly restricted to myocardial segments with WT ≤ 5 mm across all patients. This reproducibility suggests that moderate myocardial thinning represents a structurally stable substrate, whereas functional mapping tools such as CM and ILAM provide the necessary resolution to refine the precise localization of slow-conduction corridors.

### Relationship to previous studies

Our results complement and expand upon prior studies investigating the relationship between structural thinning and electrophysiological substrate in ICM.

Takigawa et al. first demonstrated that all VT isthmuses were contained within CT-defined wall-thickness channels, but only ~ 50% of such channels corresponded to functionally critical pathways [6].

Maher et al. further reported that in ICM patients, > 90% of deceleration zones on ILAM were located in regions with WT ≤ 5 mm, yet the overall anatomical overlap remained incomplete [7].

Our findings are consistent with these reports but add a functional dimension, showing that both CM-SCZ and ILAM-DZ clusters converge within WT ≈ 3–4 mm myocardium—the transition zone between electrically silent scar and viable border tissue.

Importantly, the limited correlation between WTCs and SCZ/DZs in our series underscores that CT-based anatomical channels may represent potential, not actual, conduction corridors.

Yamashita et al. previously showed that regions with WT < 5 mm are highly predictive of transmural scar and local abnormal ventricular activity (LAVA) [8].

Our data refine this concept, indicating that WT < 4 mm identifies zones requiring denser mapping and functional verification, as these areas most frequently host the deceleration and slow-conduction phenomena critical to VT maintenance, and recent outcome-oriented work further supports this integrated paradigm. In a single-center pilot of systematic CT-channel–guided ablation in post-MI VT (*n* = 39), non-inducibility after the imaging-guided pass was achieved in 48.7%, and with a mean follow-up of 47.8 ± 24.3 months, 61.9% remained VT-free; residual inducible VTs often arose either from unblocked/reconnected channels or border-zone regions outside prespecified corridors—a pattern congruent with our finding that anatomical channels nominate, but functional overlap adjudicates [9].

Platform choice may influence workflow rather than fundamentals. A recent head-to-head comparison of inHEART vs. ADAS-3D reported no significant differences in scar quantification across ischemic and non-ischemic cohorts, a trend toward higher visualization scores with ADAS-3D (particularly in non-ischemic disease), and good-to-excellent spatial correspondence (≥ 50–75%, often > 75%) between imaging-derived scar and EAM low-voltage/ablation regions in most analyzable cases. Notably, CT-derived wall thinning matched LGE-CMR enhancement in ~ 71% of cases, supporting the anatomical validity of WT-based models [10].

Finally, although MRI affords superior tissue characterization, ICD-related constraints and artifacts often limit its use [11–13]. In MRI-ineligible or suboptimal cases, delayed iodine–enhanced CT plus WT mapping provides actionable substrate surrogates and integrates cleanly with EAM.

### Clinical implications

The integration of CT-based wall-thickness maps into procedural planning offers several advantages. First, it allows pre-procedural identification of regions prone to slow conduction, helping to tailor mapping density and pacing strategies. Second, visualization of WT gradients and border-zone architecture can guide epicardial vs. endocardial access decisions.

However, our results caution against overreliance on anatomy alone. WT-derived conduction channels lack the specificity required to distinguish between conductive isthmuses and non-conductive scar extensions. Therefore, functional validation using CM and ILAM remains mandatory for precise targeting.

In practical terms, segments with WT < 4 mm should prompt higher-density mapping and detailed electrogram assessment to ensure accurate identification of slow-conduction corridors and lines of block.

### Limitations

This study has several limitations that merit consideration. The sample size was small, reflecting the exploratory nature of this multimodality integration and the limited availability of patients with high-resolution pre-procedural CT suitable for wall-thickness reconstruction. Thus, these findings should be viewed as hypothesis-generating and require validation in larger cohorts.

Spatial integration between the CT-derived geometries and the electroanatomical maps may be influenced by registration variability. Differences in arm positioning between CT acquisition (arms elevated) and the electrophysiology laboratory (arms positioned alongside the torso) can introduce subtle geometric distortions, most notably along the basal and anterolateral ventricular surfaces. Although surface registration error was ≤ 3 mm in all cases, minor misalignment cannot be fully excluded.

Variability in electroanatomical mapping density represents another methodological limitation. While several maps contained fewer points than others, all exceeded a predefined minimum threshold and underwent meticulous manual signal verification. Functional regions were annotated only when their borders were stable across two independent mapping modalities—CM vectors and ILAM isochrones—minimizing the influence of local sparsity. Although some uncertainty may persist in border areas, it is unlikely to meaningfully affect the observed anatomical–functional concordance.

Delayed iodine–enhanced CT, which provides additional tissue characterization analogous to late gadolinium enhancement on MRI, was not performed. Accordingly, wall thinning could not be directly correlated with iodine-based scar enhancement. Prior studies, however, have demonstrated that myocardial thinning alone reflects advanced structural remodeling in ischemic cardiomyopathy.

CT-derived wall-thickness maps represent a static snapshot of myocardial geometry and do not capture dynamic changes across the cardiac cycle. As a result, regional deformation or loading-dependent variability in wall thickness may not be fully represented.

Finally, all patients in this study had ischemic cardiomyopathy. The extent to which the spatial relationships between CT-derived wall thickness and functional mapping findings apply to non-ischemic substrates remains unknown and warrants further investigation.

## Conclusion

In patients with ICM, functional SC and DZ identified by CM and ILAM predominantly occur within myocardial regions exhibiting moderate wall thinning. Although WTC frequently traverse the infarct border zone, their spatial concordance with true functional isthmuses is limited. Consequently, CT-based WT maps should be viewed as an anatomical scaffold that directs focused mapping rather than as a standalone ablation guide. Our findings suggest that segments with WT < 4 mm warrant denser electroanatomical sampling and functional validation to ensure complete substrate characterization.

## Data Availability

No datasets were generated or analysed during the current study.
